# Muscle endurance, neuromuscular fatigability, and cognitive control during prolonged dual-task in people with chronic obstructive pulmonary disease: a case–control study

**DOI:** 10.1007/s00421-024-05608-x

**Published:** 2024-09-21

**Authors:** Cyril Chatain, Jean-Marc Vallier, Nicolas Paleiron, Fanny Cucchietti Waltz, Sofiane Ramdani, Mathieu Gruet

**Affiliations:** 1 Laboratoire Jeunesse-Activité Physique et Sportive-Santé (J-AP2S), Université de Toulon, La Garde, France; 2 Service de Pneumologie, Hôpital d’Instruction des Armées Saint-Anne, Toulon, France; 3 Délégation à la Recherche Clinique et à L’Innovation (DRCI), Centre Hospitalier Intercommunal de Toulon–La Seyne sur Mer (CHITS), Toulon, France; 4 Laboratoire d’Informatique, de Robotique et de Microélectronique de Montpellier (LIRMM), Université de Montpellier, Centre National de La Recherche Scientifique (CNRS), Montpellier, France

**Keywords:** Muscle functioning, COPD, Interference, Skeletal muscle, Exercise performance, N-back

## Abstract

**Purpose:**

Recent studies suggest that, compared to healthy individuals, people with chronic obstructive pulmonary disease (pwCOPD) present a reduced capacity to perform cognitive-motor dual-task (CMDT). However, these studies were focused on short-duration CMDT offering limited insight to prolonged CMDT inducing fatigue, which can be encountered in daily life. The present study aimed to explore the effect of adding a cognitive task during repeated muscle contractions on muscle endurance, neuromuscular fatigability, and cognitive control in pwCOPD compared to healthy participants.

**Methods:**

Thirteen pwCOPD and thirteen age- and sex-matched healthy participants performed submaximal isometric contractions of the knee extensors until exhaustion in two experimental sessions: (1) without cognitive task and (2) with a concurrent working memory task (i.e., 1-back task). Neuromuscular fatigability (as well as central and peripheral components measured by peripheral magnetic stimulation), cognitive performance, and perceived muscle fatigue were assessed throughout the fatiguing tasks.

**Results:**

Independently to the experimental condition, pwCOPD exhibited lower muscle endurance compared to healthy participants (*p* = 0.039), mainly explained by earlier peripheral fatigue and faster attainment of higher perceived muscle fatigue (*p* < 0.05). However, neither effect of cognitive task (*p* = 0.223) nor interaction effect (group × condition; *p* = 0.136) was revealed for muscle endurance. Interestingly, cognitive control was significantly reduced only in pwCOPD at the end of CMDT (*p* < 0.015), suggesting greater difficulty for patients with dual tasking under fatigue.

**Conclusion:**

These findings provide novel insights into how and why fatigue develops in COPD in dual-task context, offering a rationale for including such tasks in rehabilitation programs.

## Introduction

Chronic obstructive pulmonary disease (COPD) is a progressive disease characterized by airflow limitation associated with an abnormal inflammatory response of the lungs (Celli et al. 2004). In addition to its impact on lung function, COPD can also lead to systemic consequences, including neuromuscular impairments (Maltais et al. 2014) and cognitive deficits (Cleutjens et al. 2014).

Limb muscle dysfunction is well documented in people with COPD (pwCOPD) and is considered as a key systemic consequence of the disease. Some direct or indirect neuromuscular adaptations resulting from the disease have been identified (e.g., muscle fiber shift and atrophy, reduced muscle oxidative capacity, mitochondrial dysfunction), leading to muscle weakness and increased neuromuscular fatigability which is defined as a decline in an objective measure of muscle performance over a discrete period of time related to contractile and/or muscle activation (Enoka and Duchateau 2016). Additionally, neuromuscular impairments result reduced muscle endurance, corresponding to the ability of a muscle or a muscle group to sustain or perform a repeated work and resist to fatigue (Evans et al. 2015). These factors limit pwCOPD in daily life activities (Maltais et al. 2014; Gruet 2018).

PwCOPD can also exhibit impairment in cognitive processing during dual tasking (Ozsoy et al. 2021). Many daily activities involve the simultaneous accomplishment of cognitive and motor tasks, known as cognitive-motor dual-task situations (CMDT) such as talking while walking or identifying the front door key on a key ring while climbing the stairs. Although a few studies have focused on the concept of dual tasks in pwCOPD (for review, see Rassam et al. 2023), they agree that patients have insufficient attentional resources to successfully perform both tasks simultaneously compared to healthy controls, exhibiting thus a higher dual-task interference. For instance, Morlino et al. (2017) showed that the time to perform Timed Up and Go test (a basic functional mobility test) increased to a greater extent from the single-task to the dual-task condition in pwCOPD compared to healthy controls. Similarly, Heraud et al. (2018) reported that stride time variability during a 15 m walk was increased only in pwCOPD when transitioning from single to dual-task condition. These findings provide evidence that pwCOPD experience impairments in neural control during dual tasking. Although the underlying mechanisms are not fully understood, some results suggest that the greater interference observed in patients may be explained by their inability to increase oxygenation in the prefrontal cortex (Hassan et al. 2020), a brain area both involved in cognitive tasks requiring attention, memory, as well as motor planning and regulation (MacDonald et al. 2000; Tanaka and Watanabe 2012; Funahashi 2017).

While the aforementioned studies highlight the inability of pwCOPD to adequately perform short-duration dual tasks (i.e., from 10 to 30 s duration), it is yet unknown how the disease affects their capacities to perform prolonged dual tasks (i.e., involving a motor endurance task with concomitant cognitive task) which may be conducive to the development of both mental and neuromuscular fatigability.

It is well established that, in healthy individuals, the development of neuromuscular fatigue is, at least in part, related to central factors, including changes in neuromodulators concentrations in the brain and/or changes in cerebral oxygenation (Davis and Bailey 1997; Jubeau et al. 2017; Mira et al. 2020). Although there is contrasting evidence regarding the effect of mental fatigue on cerebral oxygenation (Li et al. 2009; De Wachter et al. 2024), it has been showed that the addition of a concomitant cognitive task to a motor task could impair the oxygenation of prefrontal cortex (Mehta and Parasuraman 2014), suggesting a possible interaction between the two types of fatigue. In pwCOPD, it has been showed that reduced cerebral oxygenation could alter muscle force production (Alexandre et al. 2014). However, the link between impairment in oxygen delivery to the brain and central fatigue in this population remains hypothetical (Oliveira et al. 2015).

Several studies have reported that imposing a cognitive task during a prolonged isometric contraction against a force transducer could lead to a decrease in muscle endurance (i.e., reduced time to task failure) both in young (Yoon et al. 2009; Mehta and Agnew 2012; Keller-Ross et al. 2014; Chatain et al. 2019) and old participants (Pereira et al. 2015; Shortz and Mehta 2017). For instance, Pereira et al. (2015) showed a shorter time to task failure during submaximal sustained elbow flexion in presence of high and low cognitive demand (i.e., subtraction by 13 and by 1, respectively) compared to a control condition without cognitive demand (i.e., 16 ± 8 min vs. 17 ± 4 min vs. 21 ± 7 min, respectively) in old women.

Other studies did not report any deleterious effect of the addition of cognitive demand during prolonged isometric muscle contraction on muscle endurance (Mehta and Parasuraman 2014; Guzmán-González et al. 2020). Nevertheless, these studies showed that cognitive performance was significantly decreased (i.e., increased reaction time, decreased accuracy, or both) during CMDT compared to the same cognitive task performed alone. Then, it appears that prolonged CMDT inevitably leads to a decrease in cognitive and/or motor performance compared to the equivalent motor or cognitive task performed alone.

Therefore, it seems plausible that people with impairment in neuromuscular function and cognitive processing such as pwCOPD (Dodd et al. 2010; Marillier et al. 2022) would experience even greater difficulties in performing prolonged CMDT. Prolonged CMDT are frequent in daily life activities and are also increasingly used for rehabilitation purposes to improve both motor and cognitive functions in a time-effective manner (Johansson et al. 2023; Rozenberg et al. 2023). A better understanding of the acute neuromuscular adaptations and cognitive control over the course of a prolonged fatiguing CMDT is thus essential before seeking to implement such interventions in the package of care of pwCOPD.

Accordingly, the aim of the current study was to assess the effects of adding cognitive demand (i.e., working memory task) to prolonged submaximal isometric contractions of knee extensors on muscle endurance performance (i.e., time to motor task failure) and cognitive control between pwCOPD and age- and sex-matched healthy participants. Our main hypothesis was that muscle endurance would be decreased for both groups in CMDT compared to control condition (i.e., motor task alone) and would be further decreased in pwCOPD compared to healthy controls. We also hypothesized that this larger decline in muscle endurance during CMDT in pwCOPD compared to healthy controls would be observed along with larger increase in neuromuscular fatigability and larger decrease in cognitive control.

## Methods

### Participants

Nineteen pwCOPD and seventeen age- and sex-matched control participants were initially enrolled in this study. To ensure optimal pairing between groups, we first recruited pwCOPD and then control participants with the following rule: healthy volunteers were considered eligible if their age corresponded to that of a pwCOPD of the same sex within a ± 6 year range. However, six pwCOPD and four control participants were subsequently excluded from the data analysis for various reasons, such as fatiguing task not conducted to exhaustion, or the inability to find matched pwCOPD or control participants.

Main inclusion criteria were as follows: (1) forced expiratory volume in the first second (FEV_1_; evaluated by plethysmography) < 80% of predicted values (for pwCOPD only); (2) stable state (i.e., without exacerbations) for more than 15 days (for pwCOPD only); (3) body mass index (BMI) < 30 kg.m^−2^; (4) mini-mental state examination (MMSE) score ≥ 26; (5) without known and uncontrolled chronic respiratory, cardiovascular, metabolic, renal or neuromuscular pathologies (for control participants only); (6) French participant able to provide written consent. Main non-inclusion criteria included: (1) pwCOPD treated with oral or systemic corticosteroids (> 0.5 mg.kg^−1^.day^−1^ for more than 7 days) (for pwCOPD only); (2) patient oxygen dependent (for pwCOPD only); (3) alcoholism (i.e., > 21 or 14 drinks/week for men and women, respectively); (4) psychiatric pathologies or history of behavioral disorders; (5) contraindication to peripheral nerve magnetic stimulation; (6) severe vision or hearing problems not corrected; (7) pregnant or lactating women; (8) regular vigorous physical activity with a frequency of more than three sessions per week.

This study received ethical approval from an ethic committee (CPP Ile de France III, 2019-A01986-51) and registered at www.clinicaltrials.gov (NCT04028973). All participants provided written informed consent prior to their participation in the study and were free to withdraw at any time. Each participant received financial compensation (150€) after completing the study.

### Sample size calculation

At the moment of registering the clinical trial, there were only very few studies on the topic. We believed that the best way to estimate the effect of dual-task interference in COPD came from studies focusing on the effect of COPD on the performance of a psychomotor task requiring both cognitive and motor skills (i.e., Trail Making Test). Only one case–control study provided sufficient descriptive statistics to calculate an effect size (Dogra et al. 2014). Based on the results, the minimum required sample size was calculated as 64 participants (i.e., 32 in each group) for an effect size of 0.79, a probability level of 0.05, a statistical power level of 90%, and assuming a 10% loss to follow up or study exit rate.

However, at the start of the inclusions, several results obtained from protocols closest to that of our study were published, allowing us to perform more relevant sample size calculation. This is notably the case of results from Ozsoy et al. (2021), reporting an index of cognitive dual-task interference during a muscle force production test between 35 pwCOPD and 27 healthy participants (dual-task interference = 63.2 ± 26.2% for pwCOPD and 27.4 ± 26.4% for healthy participants). Based on these results, the minimum required sample size was calculated as 24 participants (i.e., 12 in each group) for an effect size of 1.36 while keeping the same values for the other parameters (i.e., probability level, statistical power, and study exit rate described in the aforementioned a priori analysis).

In summary, the poverty of literature on the fatigability of pwCOPD in dual-task conditions makes it difficult to accurately determine the required sample size to demonstrate a significant inter-group difference between conditions for our primary outcome of interest (i.e., time to task failure). It should be noted that the initial objective was to meet the first sample size calculation (i.e., 64 participants) but in light of logistical issues, mainly caused by the COVID-19 pandemic, and the intermediate sample size calculation, we decided to stop the inclusions at 36 participants (19 pwCOPD and 17 healthy controls).

### Experimental design

This study was carried out at two research institutions. The experiments involving pwCOPD were conducted at pulmonology department of Saint-Anne army hospital (Toulon, France) while the experiments involving control participants were carried out at the University of Toulon (La Garde, France). However, all experiments were performed exactly with the same materials and environmental conditions, and by the same experimenter (C.C.).

Each participant visited the respective laboratory on three separate occasions within a 2-week period. During the first visit, anthropometric measurements were performed. Body mass was assessed using a digital weight scale (UM-076, TANITA, Tokyo, Japan) while height was measured using a stadiometer with the participants barefooted (HR001, TANITA, Tokyo, Japan). Resting oxygen saturation was recorded using a finger pulse oximeter (CMS50N, Contec Medical Systems, China). Participants were also familiarized with the study procedures and equipment and they were asked to complete several questionnaires, i.e.: (1) Mini-Mental State Examination (MMSE) questionnaire (Folstein et al. 1975); (2) the Pittsburgh Sleep Quality Index (PSQI) questionnaire (Buysse et al. 1989); (3) the French version of the Baecke questionnaire (Vol et al. 2011); (4) the Manchester COPD-fatigue scale (MCFS) (Al-shair et al. 2009); and (5) theVQ11 questionnaire (Ninot et al. 2013). The two last questionnaires were completed only by pwCOPD. Questionnaires filled out by both groups were completed using the same procedures and the same timing (i.e., ~ 30 min).

MMSE questionnaire, which includes 30 items for determining the orientation, memory, language, and visual-spatial capacities, is commonly used to assess the cognitive function. The maximum score is 30, with a score < 26 being considered to indicate mild cognitive impairments.

PSQI questionnaire was used to assess sleep quality. It comprises 19 self-reported questions providing a total score ranging from 0 (better) to 21 (worse). A total score ≥ 5 is associated with poor sleep quality.

French Baecke questionnaire was used to assess the level of habitual physical activity. It is composed of several questions about the frequency, duration, and intensity of physical activity performed in three different components (i.e., sports practice, leisure time, and daily life activities). For each component, the score ranges from 1 to 5 and the sum of these components provides the total physical activity score ranging from 3 to 15 (a high score indicating a high level of physical activity).

MCFS is a valid and reliable scale used to quantify fatigue in pwCOPD. MCFS is a 27-item self-reported scale including three dimensions (i.e., physical, cognitive, and psychosocial) to quantify the fatigue level. The total score ranges from 0 to 54 with a higher score associated with higher level of fatigue.

VQ11 is a valid French self-administered questionnaire used to assess the health-related quality of life specific to COPD and composed of 11 items distributed in three components (i.e., functional, psychological, and relational). The total score ranges from 11 to 55 with a high score reflecting a poor quality of life.

**Fig. 1:**
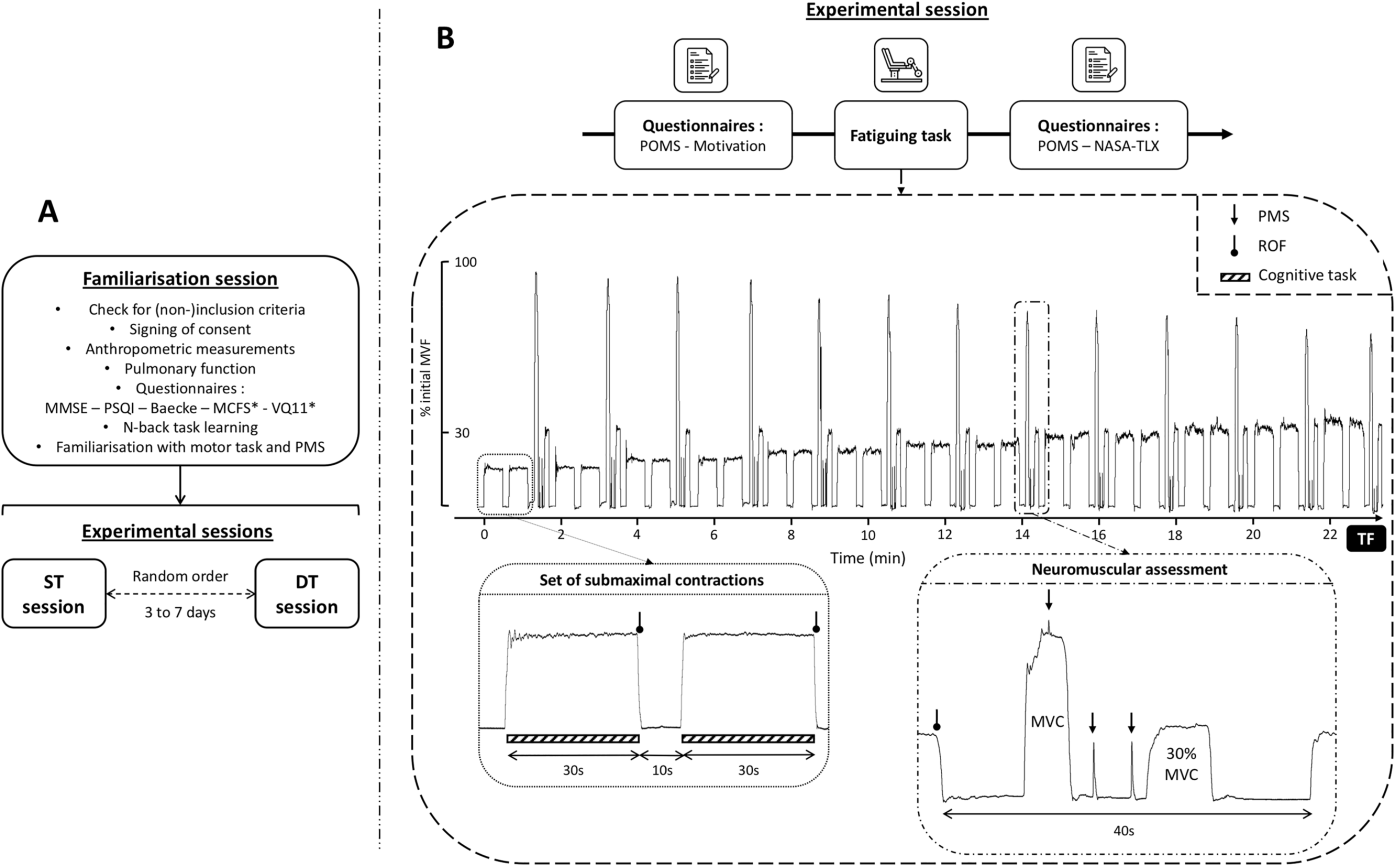
Overview of the experimental design (panel A) and the experimental sessions including the muscle fatiguing task illustrated from original recording of force signal (panel B). *MMSE* mini-mental state examination, *PSQI* Pittsburgh sleep quality index, *MCFS* Manchester COPD-fatigue scale, *VQ11* health-related quality of life, *Baecke* habitual physical activity, *MVC* maximal voluntary contraction, *MVF* maximal voluntary force, *ROF* rating-of-fatigue scale, *PMS* peripheral magnetic stimulation, *TF* task failure, *ST* single-task, *DT* dual-task, *POMS* profile of mood scale, *NASA-TLX* NASA Task load index

During the two following experimental sessions, the muscle fatiguing tasks were performed, in a random order, with or without concomitant cognitive task (Fig. 1a).

#### Experimental sessions

The two experimental sessions were: (1) a single-task (ST) session during which participants performed the muscle fatiguing task without concomitant cognitive task and (2) the dual-task (DT) session during which participants performed the same muscle fatiguing task with an added working memory task (i.e., 1-back task, see Sect. “Cognitive task”). Each session was conducted on separate days, around the same time of day for a given participant and separated by at least 72 h (maximum 7 days) to refrain from residual muscle fatigue effects. All participants were asked to refrain from caffeine and vigorous or prolonged physical activity 24 h prior to each experimental session.

Upon arrival to the experimental center, participants completed the Profile of Mood States (POMS) (Terry et al. 2003) and motivation questionnaires (Matthews et al. 2001) (Fig. 1b). For POMS questionnaire, participants were asked to rate “How are you feeling right now?” using 24 mood descriptors (e.g., depressed, sad, dynamic). For each descriptor participants had to answer using a 5-point Likert scale from 0 (not at all) to 4 (extremely). This questionnaire is divided into six subscales (i.e., fatigue, vigor, depression, confusion, tension, and anger) each containing four mood descriptors. Motivation questionnaire is composed of two scales (i.e., success and intrinsic motivation) each containing seven descriptors (e.g., “I expect the exercise to be interesting”), for which the participants had to answer using a 5-point Likert scale from 0 (not at all) to 4 (extremely).

After questionnaires completion, if the experimental session was the DT session, participants accomplished the 1-back task during 60 s. This “mental warm-up” was repeated until participants reached an accuracy ≥ 90% for one sequence of 60 s duration. 1.4 ± 0.6 sequences were performed during this “mental warm-up”. Then, a standardized warm-up of the knee extensors (i.e., 4 min 30 s intermittent isometric contractions at increasing force levels on the custom-built chair described below) was accomplished. After a 3 min recovery period, resting measurements of heart rate (HR) were obtained via a heart rate sensor (Polar H10, Polar Electro, Kempele, Finland) during 3 min.

Following HR recording, participants were asked to perform at least three maximal voluntary contractions (MVCs) of ~ 5 s duration interspaced by 1 min of recovery. If a gradual increase was observed during the first three attempts (i.e., increase > 5% between the third MVC and the largest of the first two), additional MVCs were performed to obtain a true maximal voluntary force (MVF). Strong verbal encouragements were given during each MVC to ensure maximal effort.

Then, a baseline assessment of neuromuscular function (see Sect. “Assessment of neuromuscular function”) was performed. Finally, before beginning the muscle fatiguing task, participants were asked to rate their perceived muscle fatigue using the Rating-of-Fatigue (ROF) scale composed of 11 numerical points that range from 0 (not fatigued at all) to 10 (total fatigue and exhaustion) (Micklewright et al. 2017).

At the end of each experimental session (i.e., after completion of the muscle fatiguing task), participants completed another version of POMS questionnaire. They also completed NASA-TLX questionnaire (Hart and Staveland 1988) to assess subjective workload. This questionnaire is composed of six subscales (i.e., mental demand, physical demand, temporal demand, performance, effort, and frustration) where participants had to answer from 0 (very low) to 20 (very high) for each subscale.

#### Muscle fatiguing task

Participants were set up in an adjustable custom-built chair with hips and knees at 130° (allowing optimal magnetic stimulation of the femoral nerve, e.g., Gruet et al. (2016)) and 90° of flexion, respectively. The lower right leg was attached to a force sensor (F2712 200 daN, Celians MEIRI, France) ~ 5 cm above the malleoli of the ankle joint. The muscle fatiguing task consisted in sets of intermittent submaximal isometric contractions of the right knee extensors comprising two contractions of 30 s, starting at 15% MVF for the first set and increasing by 3% MVF every two sets until task failure (Fig. 1b). Task failure (TF) was defined as the inability to reach the target force level during four consecutives seconds. The force signal feedback was displayed on a TV screen placed in front of the participants using Acqknowledge software (version 4.1, Biopac Systems, Inc., Santa Barbara, CA, USA) and recorded at 2 kHz. The participants were asked to rate their perceived muscle fatigue using the ROF scale (Micklewright et al. 2017) throughout the fatiguing task, at the end of each 30-s contraction. Heart rate was continuously recorded during the fatiguing task.

#### Assessment of neuromuscular function

Assessments of neuromuscular function were performed at baseline, at the end of each set and at TF (Fig. 1b). The timing of the assessments was standardized and performed in 40 s. Each neuromuscular assessment included a brief MVC of ~ 5 s duration performed ~ 10 s after the second 30 s submaximal contraction to allow the participants to return ROF score and the experimenter to properly position the coil for the following stimulations. During each MVC, one single superimposed stimulation was delivered to the right femoral nerve using a magnetic stimulator (Magstim200, The Magstim Company, Dyfed, UK) and a double-cone coil (50-mm outside diameter). The optimal stimulus site (i.e., position that induced the largest knee extensors twitch response) was obtained for each session and marked on the skin using a felt-tip pen to ensure reproducibility of stimulations throughout the experimental session. Following each MVC, two single stimulations were delivered on the relaxed muscle. All stimulations (i.e., search of the optimal site and neuromuscular assessment throughout the fatiguing muscle tasks) were delivered at the maximal power which can be delivered by the stimulator (i.e., 100%). Inter-day reproducibility of mechanical responses obtained from stimulations delivered on the relaxed muscle was excellent (i.e., CV = 7.6 ± 7.2%; SD = 3.0 ± 3.0N.m; ICC = 0.91). The assessment of neuromuscular function ended by a submaximal contraction of ~ 6 s performed at 30% of the baseline MVF with the aim to analyze the variability and temporal structure of force signals (Chatain et al. 2020, 2021) (data not shown as beyond the scope of this paper).

#### Cognitive task

During DT session, participants were asked to perform the muscle fatiguing task with a concomitant auditory 1-back task. We used 1-back task as stressing short-term memory is relevant to everyday life and also because we previously demonstrated (1) its feasibility in healthy subjects when used concomitantly to a motor task close to the one used in the present study (i.e., isometric quadriceps fatiguing task) and (2) that its addition to the motor task was able to induce a decline in motor performance (i.e., reduced muscle endurance), as compared to the realization of the motor task alone (Chatain et al. 2019). Participants were asked not to give a priority to motor or cognitive task and to perform both tasks as well as possible. The aim of this cognitive task was to evaluate whether a present stimulus is similar to the previous one. Stimuli were letters (i.e., J, L, M, Q, S, T, X, Z) presented using E-prime software (version 2.0, Psychology Software Tools, Sharsburg, MD, USA) and audio speakers. Participants’ responses were recorded using two computer mouses. If the current letter was similar to the previous one (i.e., match trial), the participants had to press the mouse computer held in the right hand. In reverse, if the current letter was different to the previous one (i.e., nonmatch trial), the participants had to press the mouse computer held in the left hand. Since the cognitive task was performed concomitantly to the motor task, the 1-back task was designed in such a way as to present stimuli only during the 30-s duration submaximal contractions. Each contraction comprised 14 letters presented every 2000 ms and included 4 match trials. Participants were asked to provide their answers as accurately and quickly as possible. No feedback was given between each trial or at the end of a sequence. The sequences were different from one set to another for a given participant but were the same for a given set for all participants, ensuring optimal between-subjects comparisons.

### Data analysis

#### Neuromuscular data

Time to task failure (TTF) was calculated as the time between the beginning of the first submaximal contraction and TF. MVF was considered as the peak force reached during MVC. Potentiated peak twitch (Tw_p_, i.e., twitch obtained few seconds after the completion of MVC) amplitudes were obtained from evoked responses in the relaxed muscle. The two values of Tw_p_ were averaged for each neuromuscular function assessment. The voluntary activation level (VAL) was quantified by measurement of superimposed peak twitch amplitude (Tw_s_; i.e., twitch force added during the MVC by femoral nerve stimulation) and calculated using the correction proposed by Strojnik and Komi (1998) (formula 1) including the ratio of voluntary force produced just before the superimposed stimulation (VF_b_) over the real MVF reached during MVC.

1 $$VAL(\%) = 100- {Tw}_{s}\times {(VF}_{b}/MVF)/{Tw}_{p}\times 100$$

Force signals were filtered using a low-pass digital filter with cut-off frequency of 20 Hz before computation of MVF, Tw_p_, and Tw_s_.

#### Cognitive performance

An indicator named D’, preferred to the percentage of good answer, since it is not biased by individual response tendencies (Haatveit et al. 2010), was computed to quantify the cognitive performance using the following formula:

2 $$D\prime = ZHit - ZFA$$

where Hit represents the proportion of correct matches (i.e., hits/(hits + misses)), FA (false alarms) represents the proportion of match responses on nonmatch trials (i.e., false alarms/ (false alarms + correct negative)) and Z represents the inverse of the standard normal distribution. In case of perfect scores, Hit and FA were adjusted using the formula (3) and (4), respectively

3 $$Hit =1-1/(2n)$$

4 $$FA = 1/(2n)$$

where n correspond to the number of total hits or false alarms. Accordingly, the highest possible D’ value was + 3.47, whereas the lowest value was − 3.47.

The mean reaction time and the number of non-answers were also computed for each set. Responses given with a reaction time below 100 ms, corresponding to an anticipation, were excluded from computation and were considered as non-answer.

All indicators (i.e., D’, reaction time, non-answer) were computed from two sequences of 14 letters presented during one set (i.e., from a sequence of 28 letters) to have a sufficient number of items to obtain a robust indicator of cognitive performance.

#### Heart rate and perceived fatigue

For resting measurements, HR was averaged across the central 120 s window (i.e., excluding 30 first and last seconds). For each 30 s duration submaximal contraction, HR was averaged across 10 s window centered in the middle of the contraction (i.e., excluding 10 first and last seconds). Then, two HR values obtained for both contractions composing a set were averaged to obtain one HR value for each set.

Similarly, ROF scores obtained at the end of each submaximal contraction were averaged to capture one score of perceived fatigue for each set.

#### Isotime comparisons

Since muscle fatiguing tasks were performed until task failure, and consequently had different durations between participants and conditions, our results were compared using isotime comparisons (Nicolò et al. 2019). These analyses were necessary to interpret the dynamics of different parameters at the exact same amount of work between participants and conditions.

For this purpose, for each couple of participants (i.e., one COPD patient and one sex and age-matched control participant), we selected the shortest of four fatiguing tasks as reference task allowing us to determine the different time points: (1) Pre or 0% which corresponds to measures performed before or during the first set of the fatiguing task; (2) 25% which corresponds to measures performed at quarter of the fatiguing task duration; (3) 50% which corresponds to measures performed at half of the fatiguing task duration; (4) 75% which corresponds to measures performed at three-quarter of the fatiguing task duration; (5) 100% or TF which corresponds to measures performed at TF or during the last set of the fatiguing task entirely completed. Finally, we used exactly the same sets or neuromuscular assessments for the three other fatiguing tasks. In other words, results for Pre (or 0%), 25%, 50%, 75%, and 100% time points for all parameters were compared at the same exercise time between conditions and between a COPD patient and his/her sex–age-matched control participant. Note that 100% and TF time points are only merged for the shortest of the four fatiguing tasks (i.e., for the three other tasks, TF and 100% correspond to different time points), and consequently, only comparisons for TF time point were different in terms of exercise time.

If no neuromuscular assessment or set corresponded to exactly 25%, 50%, or 75% of the duration of the shortest fatiguing task, the nearest was considered.

#### Statistical analyses

All statistical procedures were performed on SPSS (version 27.0, IBM Corp., Armonk, NY, USA). For all variables, normality was inspected using Shapiro–Wilk test. Participants’ characteristics were compared between groups using independent sample *t* test or Mann–Whitney test when conditions for the application of parametric test were not met. To detect effects of group, condition, or interaction (group × condition), TTF and scores from motivation and NASA-TLX questionnaires were compared using linear mixed models (LMMs) with one within-subject factor (condition) and one between-subject factor (group). The same statistical procedure, with one within-subject factor (time) and one between-subject factor (group), was used for indicators of cognitive control (i.e., D’, reaction time, non-answer) to detect effects of group, time or interaction (time × group). To detect effects of time, group, condition or any interactions for all other variables recorded during fatiguing tasks (i.e., neuromuscular parameters, HR, and ROF scores) and scores from the POMS questionnaire, LMMs with two within-subject factors (time and condition) and one between-subject factor (group) were used. The fixed factors of the LMMs were time, group, condition, and all interactions between these factors. For all LMMs, a random effect structured by subjects was included. If a main effect or interaction was detected, LMMs were followed by pairwise comparison tests using the Bonferroni correction. LMMs were used, because they allow to analyze repeated measures data with missing values and are robust to violations of distributional assumptions (Schielzeth et al. 2020). LMMs also provide greater statistical power that traditional analyses of variance (Ma et al. 2012). All data are presented as mean ± SD within the text and figures. Mean differences (MD) and 95% confidence intervals (95%CI) were also computed. For all analyses, statistical inferences were drawn at 0.05 level of significance.

## Results

### Subjects characteristics

Characteristics of the 26 participants (14 men and 12 women) retained for analyses are given in Table 1. No differences between groups were reported for age and anthropometric measures. PwCOPD had lower resting pulmonary function outcomes and resting pulse oxygen saturation than healthy controls, while resting HR was not different (Table 1). Regarding the neuromuscular parameters measured at rest, MVF, VAL, and Tw_p_ were not different between groups. MMSE scores were not different between groups, while scores for Baecke (lower in pwCOPD) and PSQI (higher in pwCOPD) questionnaires were significantly different. According to the PSQI cut-off score, 38% and 77% of healthy participants and pwCOPD had poor sleep quality, respectively. For questionnaires, data are presented as median ± interquartile range. For parametric and non-parametric comparisons, the effect size is expressed as Cohen’s D or Rosenthal’s r, respectively

**Table 1:** Characteristics of participants retained for analyses

	pwCOPD (*n* = 13)		CTL (*n* = 13)		*p* value		Effect size	
Variables	Absolute	% Predicted	Absolute	% Predicted	Absolute	% Predicted	Absolute	% Predicted
Anthropometric data								
Age (years)	68.6 ± 7.4	/	67.4 ± 6.6	/	*0.659*	/	*0.176*	/
Height (cm)	165.9 ± 8.4	/	171.8 ± 8.2	/	*0.085*	/	*0.704*	/
Weight (kg)	68.8 ± 10.9	/	73.9 ± 16.5	/	*0.356*	/	*0.370*	/
BMI (kg.m^−2^)	25.0 ± 3.8	/	24.9 ± 4.6	/	*0.941*	/	*0.029*	/
Cardiorespiratory data								
FEV_1_ (L)	**1.5 ± 0.5**	**57.6 ± 12.1**	**3.3 ± 0.8**	**121.2 ± 14.8**	** < *0.001***	** < *0.001***	*2.651*	*4.701*
FVC (L)	**2.5 ± 0.9**	**73.4 ± 14.9**	**4.2 ± 1.0**	**125.8 ± 17.9**	** < *0.001***	** < *0.001***	*1.851*	*3.186*
FEV_1_/FVC (%)	**62.0 ± 15.0**	**79.9 ± 18.8**	**77.5 ± 3.9**	**100.3 ± 5.5**	***0.002***	***0.001***	*1.404*	*1.466*
RV (L)	3.9 ± 2.8	196.5 ± 137.9*	/	/	/	/	/	/
TLC (L)	6.7 ± 3.0	119.8 ± 52.0*	/	/	/	/	/	/
SpO_2_ (%)	**96.6 ± 1.0**	/	**97.6 ± 1.2**	/	***0.027***	/	*0.923*	/
HR (bpm)	82.3 ± 16.3	/	73.8 ± 8.3	/	*0.108*	/	*0.654*	/
Neuromuscular data								
MVF (N.m)	138.5 ± 54.8	/	177.2 ± 59.7	/	*0.098*	/	*0.675*	/
VAL (%)	93.8 ± 3.5**	/	91.5 ± 10.4**	/	*0.971*	/	*0.170*	/
Tw_p_ (N.m)	38.0 ± 12.6**	/	43.3 ± 15.4**	/	*0.404*	/	*0.382*	/
Psychological data								
MMSE (0–30)	29.0 ± 1.0	/	28.0 ± 2.0	/	*0.687*	/	*0.083*	/
Baecke (3–15)	**6.9 ± 1.5**	/	**8.6 ± 1.4**	/	***0.014***	/	*1.044*	/
PSQI (0–21)	**7.0 ± 7.0**	/	**4.0 ± 4.0**	/	***0.002***	/	*0.586*	/
VQ11 (11–55)	27.0 ± 13.0	/	/	/	/	/	/	/
MCFS (0–54)	24.5 ± 19.5	/	/	/	/	/	/	/

Significant differences are marked in bold

*pwCOPD* people with COPD, *CTL* control participants, *BMI* body mass index, *FEV*_*1*_ forced expiratory volume in 1 s, *FVC* forced vital capacity, *RV* residual volume, *TLC* total lung capacity, *SpO*_*2*_ arterial O_2_ saturation, *HR* heart rate, *MVF* maximal voluntary force, *VAL* voluntary activation level, *Tw*_*p*_ potentiated peak twitch, *MMSE* mini-mental state examination, *Baecke* habitual physical activity, *PSQI* Pittsburgh sleep quality index, *VQ11* health-related quality of life, *MCFS* Manchester COPD-fatigue scale

*(*n* = 12)

**(*n* = 10)

P-values and effect sizes corresponding to the different variables (in absolute and %predicted values) for both pwCOPD and CTL groups are marked in italics. Significant differences between groups are highlighted in bold

### Time to task failure and force level

**Fig. 2:**
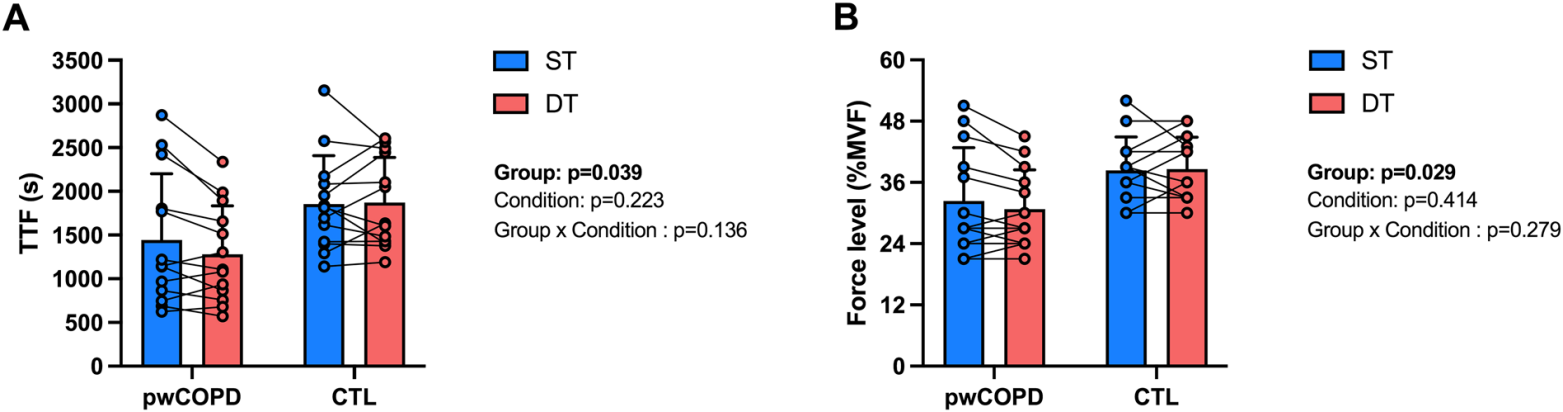
Effect of group and experimental condition on time to task failure (panel A) and force level reached (expressed in %MVF) at task failure (*n* = 26). *TTF* time to task failure, *MVF* Maximal voluntary force, *pwCOPD* people with COPD, *CTL* control participants. Data are presented as mean ± SD. Circles represent individual data

Significant main group effect [MD = 501.9; 95%CI = [28.2;975.6]; F(1,24) = 4.78; *p* = 0.039] was found for TTF without main condition effect [MD = 72.4; 95%CI = [-47.0;191.7]; F(1,24) = 1.57; *p* = 0.223] or interaction effect [F(1,24) = 2.39; *p* = 0.136] (pwCOPD ST: 1444 ± 759 s; pwCOPD DT: 1282 ± 554 s; CTL ST: 1856 ± 553 s; CTL DT 1873 ± 518 s; Fig. 2a). Significant main group effect [MD = 6.9; 95%CI = [0.8;13.1]; F(1,24) = 5.36; *p* = 0.029] was found for the force level reached at task failure without main condition effect [MD = 0.7; 95%CI = [− 1.0;2.4]; F(1;24) = 0.69; *p* = 0.414] or interaction effect [F(1,24) = 1.22; *p* = 0.279] (Fig. 2b).

### Neuromuscular fatigability

**Fig. 3:**
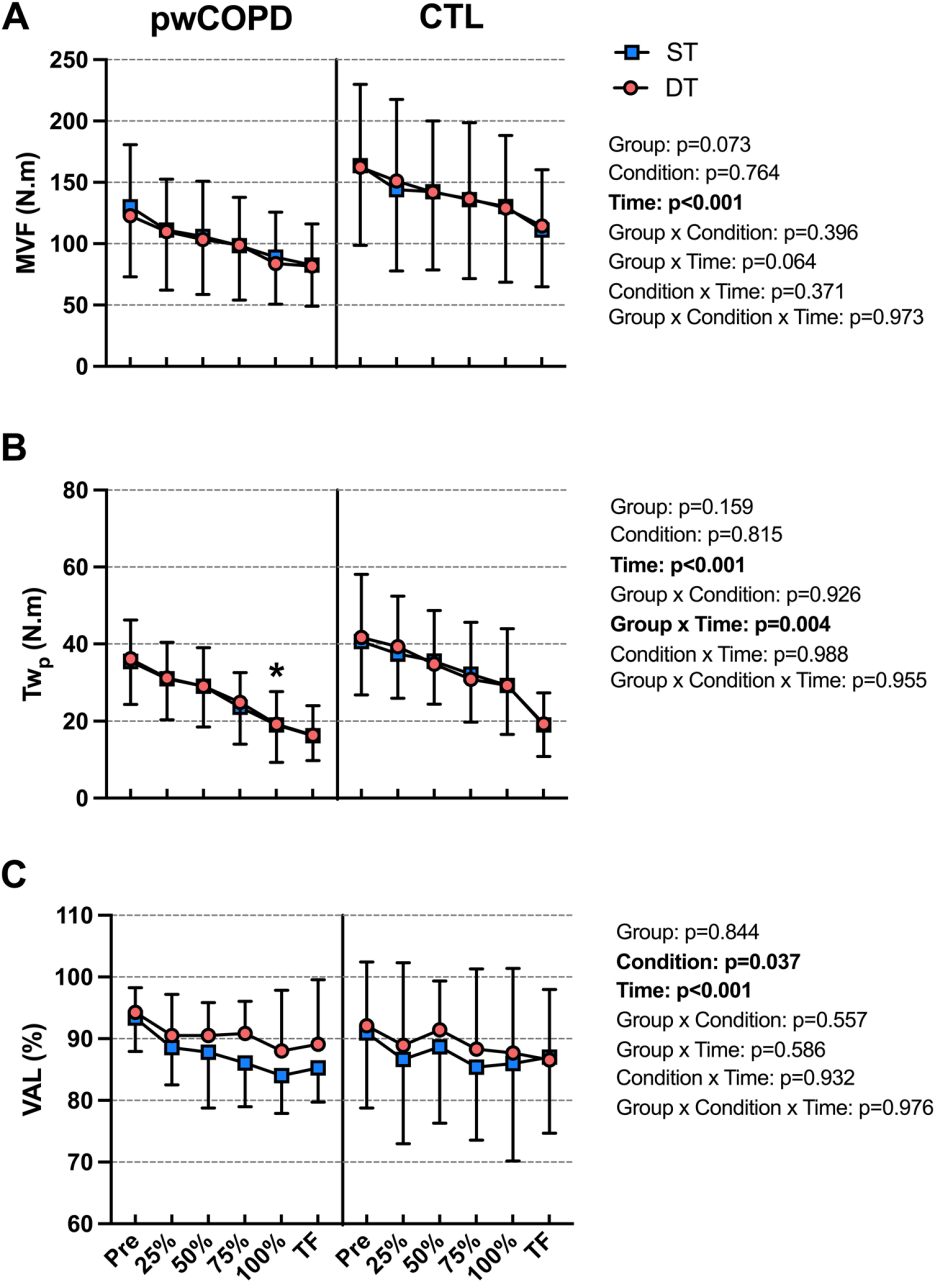
Maximal voluntary force (MVF, panel A), potentiated peak twitch (Twp, panel B), and voluntary activation level (VAL, panel C) of the knee extensors during the fatiguing tasks in people with COPD (pwCOPD) and control (CTL) participants in single-task (ST) and dual-task (DT) conditions. Pre: before the fatiguing task; 25%: at 25% of the duration of shortest test; 50%: at 50% of the duration of shortest test; 75%: at 75% of the duration of shortest test; 100%: at 100% of the duration of shortest test and TF: at task failure. Data are presented as mean ± SD (*n* = 20 for Tw_p_ and VAL while *n* = 26 for MVF). *significantly lower compared to 100% time point in CTL

MVF decreased significantly during the fatiguing tasks [(F(5,192.9) = 74.38; *p* < 0.001] without main effect of group [MD = 37.1; 95%CI = [− 3.7;77.9]; F(1,24.0) = 3.52; *p* = 0.073], condition [MD = 0.7; 95%CI = [− 5.6;4.1]; F(1,42.7) = 0.09; *p* = 0.764], or any interaction (Fig. 3a). Specifically, MVF decreased significantly from 25% time point compared to Pre time point (all *p* < 0.005). Significant main effect of time was found for Tw_p_ [F(5,145.5) = 61.4; *p* < 0.001] without main effect of group [MD = 6.5; 95%CI = [-2.8;15.8]; F(1,18.3) = 2.15; *p* = 0.159] or condition [MD = 0.3; 95%CI = [− 2.9;2.3]; F(1,27.7) = 0.056; *p* = 0.815]. More precisely, Tw_p_ decreased significantly from 25% time point compared to Pre time point (all *p* < 0.005). Significant effect was also revealed for (group × time) interaction [F(5,145.5) = 3.57; *p* = 0.004] without any other interaction. Tw_p_ was significantly lower for pwCOPD compared to CTL at 100% time point (MD = 10.2; 95%CI = [0.5;19.9]; *p*_*Bonferroni*_ = 0.04) (Fig. 3b). VAL decreased significantly during the fatiguing tasks [F(5,136.3) = 6.08; *p* < 0.001] with main condition effect [MD = 2.4; 95%CI = [0.15;4.6]; F(1,34.3) = 4.69; *p* = 0.037] and without main effect of group [MD = 0.7; 95%CI = [− 6.9;8.3]; F(1,18.0) = 0.04; *p* = 0.844] or any interaction (Fig. 3c). Specifically, VAL was significantly reduced from 75% time point compared to Pre time point (all *p* < 0.005).

### Perceived muscle fatigue

**Fig. 4:**
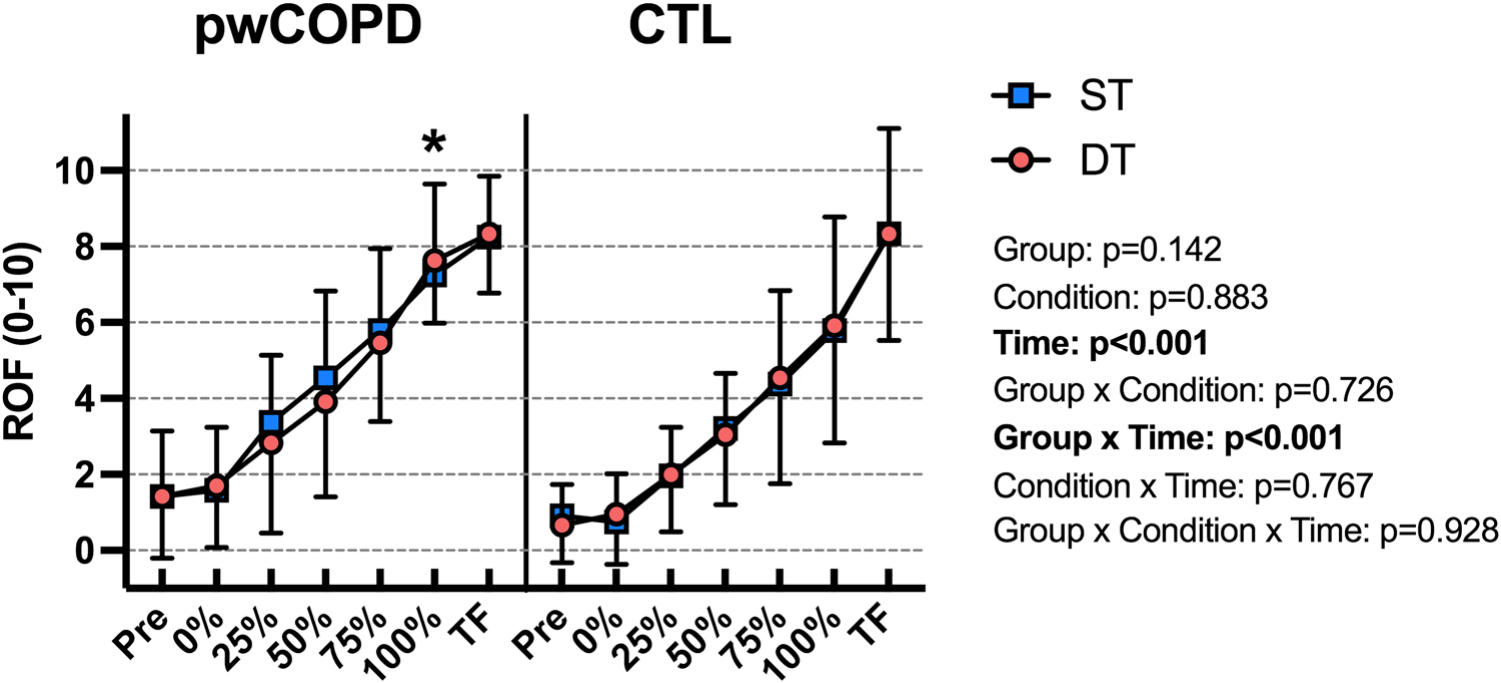
Rating-of-fatigue (ROF) scores during the fatiguing task in people with COPD (pwCOPD) and control (CTL) participants in single-task (ST) and dual-task (DT) conditions. Pre: before the fatiguing task; 0%: at the end of the first set of fatiguing task; 25%: at the end of set corresponding to 25% of the duration of shortest test; 50%: at the end of set corresponding to 50% of the duration of shortest test; 75%: at the end of set corresponding to 75% of the duration of shortest test; 100%: at the end of set corresponding to 100% of the duration of shortest test and TF: at task failure. Data are presented as mean ± SD (*n* = 24). *significantly higher compared to 100% time point in CTL

ROF score increased significantly throughout the fatiguing tasks [F(6,216.6) = 162.7; *p* < 0.001] without main effect of group [MD = 0.8; 95%CI = [− 0.3;2.0]; F(1,22.8) = 2.32; *p* = 0.142] or condition [MD = 0.04; 95%CI = [− 0.57;0.49]; F(1,40.9) = 0.02; *p* = 0.883]. Significant effect was revealed for (group × time) interaction [F(6,216.6) = 5.61; *p* < 0.001] without any other interaction. Encompassing both conditions, ROF score was significantly higher for pwCOPD at 100% time point (MD = 1.6; 95%CI = [0.3;2.9]; *p*_*Bonferroni*_ = 0.017) (Fig. 4).

### Cognitive performance

**Fig. 5:**
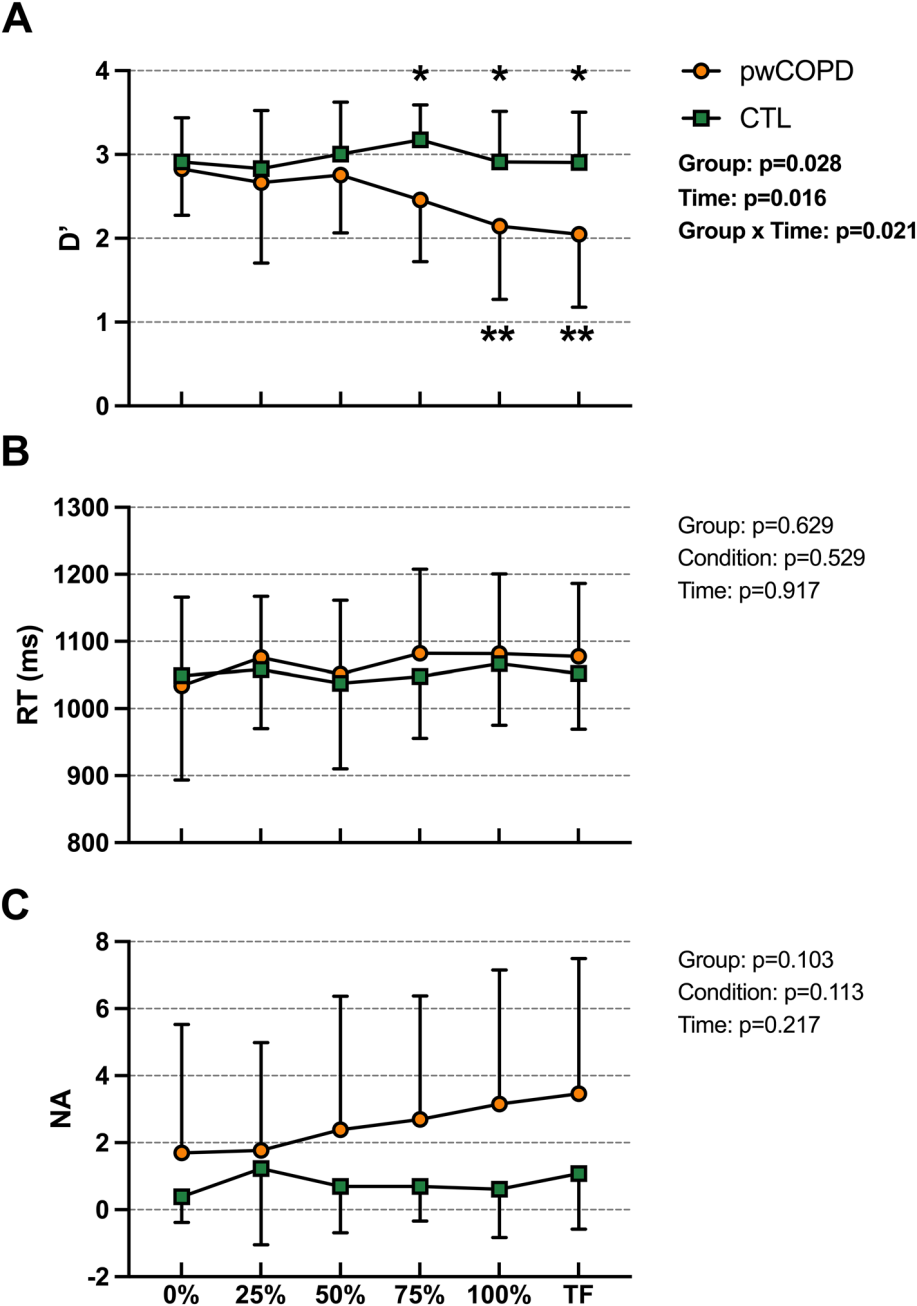
D’ (panel 1), reaction time (RT, panel B) and number of non-answers (NA, panel C) for 1-back task performed during the dual-task condition for people with COPD (pwCOPD) and control (CTL) participants. 0%: during the first set of the fatiguing task; 25%: at set corresponding to 25% of the duration of shortest test; 50%: at set corresponding to 50% of the duration of shortest test; 75%: at set corresponding to 75% of the duration of shortest test; 100%: at set corresponding to 100% of the duration of shortest test and TF: at the last set entirely completed. Data are presented as mean ± SD (*n* = 26).*significant difference between pwCOPD and CTL; **significant difference with 0% time point in pwCOPD

D’ decreased significantly throughout the fatiguing tasks [F(5,69.5) = 3.01; *p* = 0.016] with main group effect [MD = 0.47; 95%CI = [0.06;0.89]; F(1,24.0) = 5.46; *p* = 0.028] and interaction effect [F(5,69.5) = 2.87; *p* = 0.021]. D’ was significantly lower for pwCOPD at 75% (MD = − 0.72; 95%CI = [− 1.26;− 0.17]; *p*_*Bonferroni*_ = 0.011), 100% (MD = -0.77; 95%CI = [− 1.31;− 0.22]; *p*_*Bonferroni*_ = 0.006) and TF (MD = -0.86; 95%CI = [-1.40;-0.31]; *p*_*Bonferroni*_ = 0.002) time points (Fig. 5a). LMM revealed no significant changes of reaction time throughout the fatiguing tasks [F(5,69.4) = 0.836; *p* = 0.529], with no significant effect of group [MD = 15.2; 95%CI = [-79.1;48.8]; F(1,24.8) = 0.239; *p* = 0.629] or interaction [F(5,69.4) = 0.29; *p* = 0.917] (Fig. 5b). No significant main time effect [F(5,70.7) = 1.86; *p* = 0.113], main group effect [MD = 1.74; 95%CI = [-0.38;3.87]; F(1,24.1) = 2.87; *p* = 0.103], or interaction effect [F(5,70.7) = 1.45; *p* = 0.217] was found for the number of non-answers (Fig. 5c).

### Heart rate

**Fig. 6:**
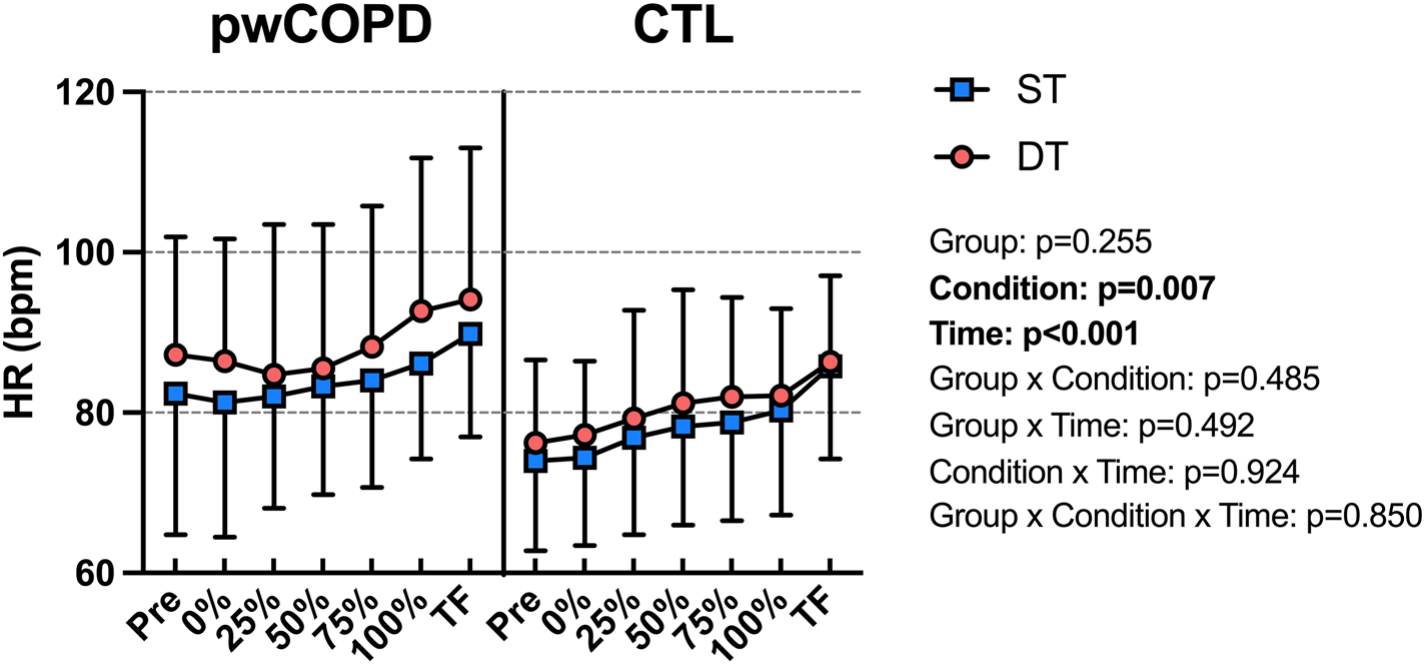
Heart rate (HR) during the fatiguing task for people with COPD (pwCOPD) and control (CTL) participants during single-task (ST) and dual-task (DT) conditions. Pre: before the fatiguing task; 0%: during the first set of fatiguing task; 25%: during the set corresponding to 25% of the duration of shortest test; 50%: during the set corresponding to 50% of the duration of shortest test; 75%: during the set corresponding to 75% of the duration of shortest test; 100%: during set corresponding to 100% of the duration of shortest test and TF: during the last set entirely completed. Data are presented as mean ± SD (*n* = 24)

HR increased significantly throughout the fatiguing tasks [F(6,208.9) = 15.3; *p* < 0.001] with main condition effect [MD = 3.1; 95%CI = [0.9;5.3]; F(1,44.0) = 8.01; *p* = 0.007] and without main effect of group [MD = 6.2; 95%CI = [− 4.8;17.2]; F(1,22.06) = 1.37; *p* = 0.255] or any interaction (Fig. 6).

### Questionnaires

#### Motivation

Intrinsic motivation was not significantly different between groups [MD = 0.73; 95%CI = [− 1.6;3.1]; F(1,24) = 0.41; *p* = 0.528], conditions [MD = 0.58; 95%CI = [− 0.4;1.5]; F(1,24) = 1.55; *p* = 0.225] with the absence of interaction effect [F(1,24) = 1.17; *p* = 0.291] (pwCOPD ST: 21.2 ± 2.3; pwCOPD DT: 20.1 ± 3.9; CTL ST: 21.4 ± 3.0; CTL DT 21.3 ± 3.2). In the same way, success motivation was not different between groups [MD = 0.15; 95%CI = [-3.5;3.2]; F(1,24) = 0.01; *p* = 0.924], conditions [MD = 0.39; 95%CI = [− 1.0;1.8]; F(1,24) = 0.32; *p* = 0.579] and without effect of interaction [F(1,24) = 0.01; *p* = 0.911] (pwCOPD ST: 15.6 ± 4.4; pwCOPD DT: 15.3 ± 5.7; CTL ST: 15.5 ± 3.2; CTL DT 15.1 ± 4.1).

#### Mood

All detailed results for POMS questionnaire are available in Table 2. In short, three-way mixed model revealed a significant main group effect for anger [MD = 0.8; 95%CI = [0.04;1.58]; F(1,24.0) = 4.7; *p* = 0.04], fatigue [MD = 1.6; 95%CI = [0.1;3.2]; F(1,24.6) = 4.63; *p* = 0.041], and tension [MD = 1.1; 95%CI = [0.07;2.2]; F(1,24.0) = 4.8; *p* = 0.038] subscales, with scores systematically higher for pwCOPD. Main time effect was showed for depression [MD = 0.4; 95%CI = [0.1;0.7]; F(1,32.3) = 7.53; *p* = 0.01], fatigue [MD = 1.5: 95%CI = [1.0;2.1]; F(1,58.4) = 30.4; *p* < 0.001], tension [MD = 0.7; 95%CI = [0.2;1.2]; F(1,39.5) = 7.35; *p* = 0.01], and vigor [MD = 1.6; 95%CI = [0.9;2.3]; F(1,65.8) = 20.5; *p* < 0.001] subscales, with score of fatigue significantly increased after the fatiguing tasks while a significant decrease was revealed for scores of depression, tension, and vigor. Main condition effect was revealed for confusion [MD = 0.7; 95%CI = [0.3;1.1]; F(1,38.6) = 10.8; *p* = 0.002] and tension [MD = 0.9; 95%CI = [0.2;1.4]; F(1,36.7) = 8.09; *p* = 0.007] subscales, with scores significantly higher in DT than in ST condition.

**Table 2:** Scores of Profile of Mood States questionnaire (POMS) before (Pre) and after (Post) single (ST) and dual (DT) fatiguing tasks in people with COPD (pwCOPD) and control participants (CTL)

		pwCOPD		CTL		*p* value	
		ST	DT	ST	DT		
Anger	Pre	0.0 ± 0.0 *[0.0–3.0]*	0.0 ± 3.0 *[0.0–8.0]*	0.0 ± 0.0 *[0.0–0.0]*	0.0 ± 0.0 *[0.0–0.0]*	*Time*	*0.069*
						*Group*	***0.040***
	Post	0.0 ± 0.0 *[0.0–2.0]*	0.0 ± 0.0 *[0.0–6.0]*	0.0 ± 0.0 *[0.0–0.0]*	0.0 ± 0.0 *[0.0–0.0]*	*Condition*	*0.101*
						*Interaction*	none
Confusion	Pre	0.0 ± 2.0 *[0.0–6.0]*	2.0 ± 4.0 *[0.0–7.0]*	0.0 ± 0.0 *[0.0–2.0]*	0.0 ± 2.0 *[0.0–2.0]*	*Time*	*0.318*
						*Group*	*0.084*
	Post	0.0 ± 1.0 *[0.0–6.0]*	1.0 ± 3.0 *[0.0–8.0]*	0.0 ± 0.0 *[0.0–3.0]*	0.0 ± 2.0 *[0.0–2.0]*	*Condition*	***0.002***
						*Interaction*	none
Depression	Pre	0.0 ± 1.0 *[0.0–7.0]*	1.0 ± 2.0 *[0.0–8.0]*	0.0 ± 0.0 *[0.0–2.0]*	0.0 ± 0.0 *[0.0–0.0]*	*Time*	***0.010***
						*Group*	*0.070*
	Post	0.0 ± 0.0 *[0.0–3.0]*	0.0 ± 0.0 *[0.0–6.0]*	0.0 ± 0.0 *[0.0–0.0]*	0.0 ± 0.0 *[0.0–0.0]*	*Condition*	*0.060*
						*Interaction*	***#-##***
Fatigue	Pre	1.0 ± 4.0 *[0.0–6.0]*	3.0 ± 4.0 *[0.0–11.0]*	0.0 ± 1.0 *[0.0–3.0]*	0.0 ± 1.0 *[0.0–4.0]*	*Time*	** < *0.001***
						*Group*	***0.041***
	Post	3.0 ± 3.0 *[0.0–9.0]*	5.0 ± 4.0 *[0.0–12.0]*	2.0 ± 4.0 *[0.0–6.0]*	2.0 ± 3.0 *[0.0–7.0]*	*Condition*	*0.164*
						*Interaction*	none
Tension	Pre	0.0 ± 2.0 *[0.0–4.0]*	2.0 ± 5.0 *[0.0–8.0]*	0.0 ± 1.0 *[0.0–4.0]*	0.0 ± 1.0 *[0.0–5.0]*	*Time*	***0.010***
						*Group*	***0.038***
	Post	0.0 ± 1.0 *[0.0–3.0]*	0.0 ± 2.0 *[0.0–11.0]*	0.0 ± 0.0 *[0.0–2.0]*	0.0 ± 0.0 *[0.0–1.0]*	*Condition*	***0.007***
						*Interaction*	***##***
Vigor	Pre	8.0 ± 5.0 *[0.0–13.0]*	9.0 ± 3.0 *[0.0–16.0]*	9.0 ± 3.0 *[4.0–16.0]*	9.0 ± 6.0 *[4.0–16.0]*	*Time*	** < *0.001***
						*Group*	*0.487*
	Post	7.0 ± 4.0 *[0.0–15.0]*	9.0 ± 6.0 *[2.0–15.0]*	8.0 ± 5.0 *[1.0–13.0]*	9.0 ± 6.0 *[1.0–15.0]*	*Condition*	*0.332*
						*Interaction*	none

Data are presented as median ± interquartile range and total range [min–max] (*n* = 26)

Significant differences are marked in bold

^#^Significant (time x group) interaction effect

^##^Significant (condition x group) interaction effect

For each item, ranges [min-max] are marked in italics. P-values for different main or interaction effects from LMMs are marked in italics with significant effects highlighted in bold

#### NASA-TLX

LMM revealed that overall score from NASA-TLX questionnaire was significantly higher after DT than after ST condition [MD = 6.5; 95%CI = [0.4;12.6]; F(1,24) = 4.79; *p* = 0.039] without main group effect [MD = 6.0; 95%CI = [− 5.7;17.7]; F(1,24) = 1.13; *p* = 0.299] or interaction effect [F(1,24) = 1.03; *p* = 0.320] (pwCOPD ST: 37.8 ± 14.2; pwCOPD DT: 47.3 ± 19.0; CTL ST: 34.8 ± 13.7; CTL DT 38.3 ± 17.5). Mental demand was significantly higher after DT than after ST condition [MD = 2.5; 95%CI = [0.6;4.4]; F(1,24) = 7.27; *p* = 0.013] without main group effect [MD = 0.3; 95%CI = [− 2.2;2.9]; F(1,24) = 0.08; *p* = 0.785] or interaction effect [F(1,24) = 0.002; *p* = 0.967] (pwCOPD ST: 3.1 ± 3.5; pwCOPD DT: 5.5 ± 5.1; CTL ST: 2.7 ± 3.0; CTL DT 5.2 ± 4.1). Temporal demand was significantly higher after DT than after ST condition [MD = 2.9; 95%CI = [1.1;4.8]; F(1,24) = 10.63; *p* = 0.003] without main group effect [MD = 0.4; 95%CI = [-2.7;3.5]; F(1,24) = 0.06; *p* = 0.801] or interaction effect [F(1,24) = 0.60; *p* = 0.447] (pwCOPD ST: 4.2 ± 3.8; pwCOPD DT: 7.8 ± 5.3; CTL ST: 4.5 ± 4.5; CTL DT 6.7 ± 4.2). No main condition, group, or interaction effect was found for physical demand, performance, effort, and frustration subscales.

## Discussion

The current study aimed at evaluating the impact of adding a working memory task during prolonged submaximal isometric contractions of knee extensors on muscle endurance and the associated modulations in neuromuscular function, cognitive control, and perceived fatigue in pwCOPD compared to age- and sex-matched healthy participants. To our knowledge, this is the first study to explore muscle endurance and the associated neuromuscular adjustments in pwCOPD in dual-task context, providing novel insights into how and why fatigue develops in COPD in situations close to those which can be encountered in daily life. Contrary to our initial hypothesis, adding a low concurrent cognitive demand (i.e., 1-back task) to prolonged isolated muscle exercise did not decrease muscle endurance in both pwCOPD and healthy participants. Nevertheless, while there was no significant difference in TTF between single- and dual-task conditions, pwCOPD exhibited a decrease in cognitive performance at the end of the fatiguing dual-task contrary to healthy participants who were able to maintain cognitive control despite progressive development of neuromuscular fatigability. This finding suggests an exacerbated dual-task interference under fatigue state in pwCOPD.

### Effect of COPD on muscle endurance

As expected, regardless of the experimental conditions, pwCOPD showed reduced TTF compared to healthy participants. This result corroborates the decrease in muscle endurance in pwCOPD reported in previous studies (for review, see Evans et al. 2015). Our comprehensive assessment of fatigue and its potential contributors provide further insights on the mechanisms underlying lower muscle endurance in COPD.

In its contemporary interpretation (Enoka and Duchateau 2016), fatigue arises from the emergence and interaction of two primary forms of fatigability: (1) performance fatigability, which encompasses the peripheral and central factors underlying reduced muscle performance (e.g., calcium kinetics, voluntary activation) that can be objectively assessed, and (2) perceived fatigability, which is driven by homeostatic and psychological factors and that is often quantified using scales or questionnaires. In accordance with this model (and its adapted version in people with chronic respiratory disorders (Gruet 2018)), several factors could explain the reduced TTF in pwCOPD reported in our study.

First, our results suggest that the reduction in muscle endurance in pwCOPD is, at least in part, caused by peripheral muscle mechanisms, as revealed by the early decrease in Tw_p_ amplitude (i.e., significant reduced Tw_p_ amplitude in pwCOPD compared to healthy participants at 100% time point), certainly related to the skeletal muscle pathophysiology of the disease (e.g., decreased percentage of aerobic fibers, reductions in the activity of oxidative enzymes and capillary density, oxidative stress, early lactate release) (Gea et al. 2013). The importance of peripheral parameters is also indirectly confirmed by the absence of between-group differences for the kinetics of central fatigue (i.e., similar changes in VAL). While reduced central drive has been proposed as a limiting factor to physical exertion in various cardiorespiratory disorders (Marillier et al. 2022), it is of note that most evidence is derived from studies including severe patients (e.g., Marillier et al. 2018, 2022), with thus increasing likeliness of a role of chronic hypoxemia inducing cerebral impairments that are exacerbated during physical exercise. The role of central factors in limiting exercise performance is probably much more limited in moderate severity profile. For instance, our findings are in line with a previous study showing similar kinetics of central fatigue during quadriceps isometric exercise in mild-to-moderate cystic fibrosis compared to healthy controls (Gruet et al. 2016).

Considering the neuromuscular impairments (notably peripheral) induced by COPD (Maltais et al. 2014; Marillier et al. 2021), we might have expected a more important difference between groups, and earlier onset during the fatiguing tasks (e.g., at 50% and/or 75% time points) for Tw_p_ amplitude. The small observed difference (i.e., only at 100% time point) can be explained by the notion of critical torque which corresponds to the theoretical maximum force that can be maintained without fatigue. Although sometimes overestimated (Hendrix et al. 2009), the critical torque corresponds to ~ 30% MVF for intermittent isometric tasks similar to the one used in the present study (for review, see Poole et al. 2016). Given the force levels reached at the end of the fatiguing tasks (i.e., 31.6 ± 9.0% MVF for pwCOPD and 39.0 ± 6.3% MVF for healthy participants), we can consider that a large part of submaximal fatiguing contractions was performed below the critical torque, which may have contributed to reduce the differences in neuromuscular fatigability between groups.

Our results also revealed a faster attainment of an important perceived muscle fatigue in pwCOPD in comparison with control participants. It is of note that we did not measure perceived exertion in the present study to limit the number of scores and avoid the risk of scores contamination, potentially decreasing the accuracy of each rating (Gruet et al. 2018; Lewthwaite et al. 2021). However, as effort perception is one of the main drivers of perceived muscle fatigue (Behrens et al. 2023) and because both are very closely related during motor exercise inducing progressive fatigue development (Micklewright et al. 2017), we might expect higher effort perception in pwCOPD close to task failure, as compared to healthy controls. Previous studies in healthy participants using similar exercise modalities (e.g., low-intensity sustained isometric contractions of the knee extensors) demonstrated that task failure was related to earlier attainment of maximal perceived effort (Souron et al. 2020). Collectively, these findings suggest that higher perceived muscle fatigue/effort may have contributed to earlier disengagement from the motor task in pwCOPD, explaining, at least in part, their lower TTF. The fact that this earlier disengagement had no effect on MVF and VAL (i.e., no further decrease for these parameters in pwCOPD at TF compared to healthy participants) could be explained by: i) the time between the end of the last submaximal fatiguing contraction (i.e., TF) and the accomplishment of the last MVC (i.e., last neuromuscular assessment) of approximately 10 s, that could allow a rapid recovery masking difference between pwCOPD and healthy participants and ii) the fact that participants knew that the fatiguing task was finished, motivating them to produce a higher effort to perform the last MVC.

Moreover, outcomes from the POMS questionnaire suggest more elevated levels of anger, fatigue, and tension among pwCOPD compared to healthy controls. It is of note that beyond the numerous pathophysiological factors that may contribute to the higher muscle susceptibility to fatigue in COPD (see Maltais et al. (2014) for review), lower physical activity levels (supported by Baecke questionnaire results) and compromised sleep quality (supported by PSQI questionnaires results) may have also played a role through their negative influence on various modulating factors of both perceived (e.g., wakefulness, arousal) and performance fatigability (e.g., force capacity) (Enoka and Duchateau 2016; Gruet 2018).

Hence, it appears that the reduced TTF observed in pwCOPD is attributed to a combination of psychological and physiological factors.

### Effect of CMDT on muscle endurance

Contrary to our expectations, we did not identify a difference in TTF between ST and DT conditions, for both pwCOPD and healthy individuals. This finding is in contrast to the results reported by Chatain et al. (2019) in young healthy adults, showing a reduced muscle endurance when participants performed a 1-back task concomitantly to a motor task relatively close to that used in the present study (i.e., intermittent submaximal contractions of knee extensors performed at 20% MVF until exhaustion). Nevertheless, differences in protocols characteristics could explain the discrepancies in the results.

The duration of muscle contractions performed during motor task in Chatain et al. (2019) was longer than in the present study (i.e., sets of contractions of 170 s vs. 70 s in the present study), for identical neuromuscular evaluation timings (i.e., 40 s) during which no cognitive task was performed. Therefore, participants were exposed to the 1-back task 75% of the exercise time in Chatain et al. (2019), compared to 55% in the present study. This reduced time exposure to the cognitive task may have impede the development of mental fatigue, which has been suggested as one of the main contributors to the decline in muscle endurance in CMDT (Chatain et al. 2019). This assumption is reinforced by the scores obtained from the mental demand subscale of the NASA-TLX questionnaire which, although significantly different between ST and DT, remain relatively low compared to other studies (Shortz and Mehta 2017; Chatain et al. 2019). It is of note that participants started the DT condition with a level of cognitive performance lower that the maximum achievable (i.e., D'_0%_ = 2.87 vs. D'_max_ = 3.47, i.e., 83% of maximum cognitive performance). Thus, from the beginning of the task, participants were not able to perform the cognitive task maximally, probably to focus on the motor task, which could mask the detrimental effects of the cognitive task on muscle endurance.

One may believe that a more demanding level of n-back task (e.g., 2-back) would have been more appropriate to alter muscle endurance. For instance, Csipo et al. (2021) showed that a 2-back task resulted in increased neurovascular coupling while 1-back task did not, compared to a control task (i.e., 0-back). However, the absence of specific neurophysiological adjustments during 1-back performed in isolation may not result in the absence of effect when performed concomitantly with a motor task. It is of note that we observed a higher HR during DT compared to ST in our study supporting this idea. Moreover, results observed in young adults may not be translate to older populations. It is likely that a 2-back task would have been too difficult for our population to be completed in a dual-task situation.

While in one hand the cognitive load was not sufficient to have detrimental effect on endurance time, in the other hand, the cognitive task was enough difficult to reveal the inability of pwCOPD to maintain cognitive performance through the endurance task as they probably allocate more cerebral resources to maintain motor performance.

It should be stressed that VAL was significantly lower during ST compared to DT condition. A hypothesis to explain this intriguing result could be the installation of a slightly greater state of boredom in ST compared to DT condition. It seems likely that the concomitant simple cognitive task (i.e., 1-back) makes the fatiguing task in DT condition more challenging, less monotonous and potentially served as a distracting stimulus allowing participants to maintain a greater motivation level (a crucial feature to accomplish maximal effort) during MVCs. In line with our initial hypothesis (i.e., reduced endurance time in DT compared to ST condition), it also seems plausible that without this hypothetical state of boredom, participants could have reached a higher VAL in ST condition (i.e., similar to DT condition), which could have resulted in a slightly higher MVF allowing participants to have a longer endurance time in ST condition. Nevertheless, the complexity of the interaction between fatigue, motivation, boredom, and weariness makes it difficult to interpret this result, and further studies are needed to better understand the effect of a simple concomitant cognitive task on these psychological parameters.

### Effect of CMDT and COPD on muscle endurance

Despite the absence of an (group x condition) interaction effect on muscle endurance, we observed an average TTF reduction in DT condition of − 11.2% among pwCOPD, while it showed a slight increase of 0.9% in healthy participants.

Beyond the aforementioned questionable engagement of the participants in the cognitive task, the absence of a statistically significant interaction effect could be attributed to an important interindividual variability in endurance times or also explained by the significant decrease of cognitive performance observed at the end of the fatiguing task in pwCOPD compared to controls. Indeed, the D’ values decreased significantly only in pwCOPD from 100% time point. It is of note that this decrease in cognitive performance occurred despite unchanged reaction time and non-answer, suggesting that they do not deliberately give up on the cognitive task (which may have resulted in a decrease in reaction time and/or an increase in number of non-answers) but probably allocate more resources to the maintenance of the motor task. Therefore, it can be hypothesized that if pwCOPD would have maintained a constant level of cognitive performance, this might have compromised the motor task, leading to decreased muscle endurance in DT condition. In any case, the reduced cognitive performance of pwCOPD at the end of prolonged CMDT reflects a higher level of interference, corroborating the data from previous work in brief unfatiguing tasks (Morlino et al. 2017; Heraud et al. 2018; Ozsoy et al. 2021) and extending this observation in a fatiguing context.

Nevertheless, while cognitive performance was reduced in pwCOPD in the presence of muscle fatigue, our results showed that without fatigue (i.e., during the first steps of the fatiguing tasks), pwCOPD exhibited similar DT performance compared to healthy participants (i.e., similar performance in the 1-back task while maintaining similar submaximal relative force), contrasting with the previous studies (Morlino et al. 2017; Heraud et al. 2018; Ozsoy et al. 2021). This result can be explained by the fact that the motor task used in the current study is based on isolated muscle contractions, while the previous studies used whole-body tasks (e.g., walking task, TUG test). This finding suggests that in the absence of fatigue, the presence of motor dual-task interference is directly related to the nature of the motor and/or cognitive task. Considering this point and our results showing that fatigue can exacerbate dual-task interference for pwCOPD, we can hypothesize that the effects of fatigue could be even greater when DT involves a whole-body motor task.

### Clinical implications

Both cognitive function and physical fitness are impaired in pwCOPD (Dodd et al. 2010; Maltais et al. 2014). Exercise training are already included in the package of care of pwCOPD with accumulating evidence regarding positive effect on both neuromuscular function and cardiorespiratory fitness (Spruit et al. 2016). Meanwhile, interest for cognitive training is more recent, with very few trials (Incalzi et al. 2008; van Beers et al. 2021) showing only limited benefits when performed in isolation (e.g., unchanged psychological well-being and healthy lifestyle goals following 12-week of working memory training (van Beers et al. 2021)). It is also unclear whether training motor and cognitive functions in isolation may translate in improvements in cognitivo-motor processing during dual tasking, and ultimately improve ability to better perform daily living tasks. Moreover, perceived lack of time or competing priorities are important barrier to structured physical activity among older adults (Kosteli et al. 2016), including pwCOPD and notably those in full-time employment (Kosteli et al. 2017). In this context, CMDT training may (1) serve as a time-effective strategy to improve both motor and cognitive functions and (2) present the advantage to tap into cerebral resources that are not fully engaged when the two tasks are performed independently, as suggested by the exacerbated dual-task interference under fatigue state in pwCOPD.

Our results also provide evidence on the feasibility of adding a cognitive challenge during prolonged fatiguing physical exercise in pwCOPD. It is interesting to observe that the addition of a cognitive task, allowing a cognitive training, did not lead to a premature disengagement from the motor task and differences in peripheral fatigability, since producing contractile fatigue is a prerequisite for optimal results from an exercise training program (e.g., Burtin et al. 2012). In other words, implementing a cognitive task in conjunction with a motor task may not reduce the benefits associated with a fatiguing motor task used in a context of motor training. This may be particularly interesting for resistance training, which is an important component of exercise rehabilitation in COPD (Spruit et al. 2002; Gloeckl et al. 2013) aiming to increase muscle strength and endurance while keeping ventilatory requirements tolerable. Our results are particularly relevant in this context as the working memory task was added to a single-joint exercise of the most targeted muscle group (i.e., quadriceps) in resistance training sessions in COPD. However, it is of note that implementing cognitive task to whole-body exercise (e.g., aerobic cycling exercise) may be difficult as increased dyspnea perception may compromise, at some points during the session, the ability to focus on the concomitant cognitive task.

Finally, our findings suggest that adding cognitive challenge does not compromise the motor task in terms of muscle fatigability and task duration which are important components to elicit muscle adaptations while adding a concurrent cognitive challenge. Of course, the exact difficulty and duration of both cognitive and motor tasks remain to be adjusted for real exercise training sessions.

### Limitations

A first limitation concerns the type of fatiguing task used in the present study (i.e., incremental intermittent isometric muscle contractions). Previous studies reporting a deleterious effect of adding a cognitive task on muscle endurance (Yoon et al. 2009; Mehta and Agnew 2012; Keller-Ross et al. 2014; Pereira et al. 2015; Chatain et al. 2019) used constant intensity isometric muscle contractions (sustained or intermittent). It seems highly likely that the effect of dual task on endurance performance is task dependent. Therefore, the use of incremental fatiguing task might have obscured the differences in time to task failure between ST and DT conditions.

Another limitation concerns the use of peripheral stimulation which does not seem to be the most sensitive method to detect small changes in VAL. For instance, Alexandre et al. (2020) highlighted a reduced VAL in pwCOPD compared to healthy participants using transcranial magnetic stimulation, while no difference was found using peripheral magnetic stimulation.

Finally, most pwCOPD included in the present study had moderate airflow limitation severity, restricting interpretation of the effect of the disease severity on the level of interference during fatiguing dual-task.

### Conclusion

In conclusion, our study revealed that COPD did not induce a larger decrease in muscle endurance in the presence of concomitant cognitive task compared to a control condition. Although neuromuscular fatigability was not affected by the addition of cognitive demand, pwCOPD showed reduced cognitive performance at the end of the fatiguing dual-task, reflecting an increase of interference in a fatigue situation for these patients. These findings highlight the importance of considering the integration of fatiguing CMDT in rehabilitation programs as a strategy to improve cognitive function while allowing to generate a sufficient fatigue level to induce neuromuscular adaptations. Nevertheless, more research is needed to fully understand COPD effect on fatigability during prolonged dual-task, the underlying mechanisms involved and the interest of including these procedures in rehabilitation programs.

## Data Availability

Data can be obtained from the first author (Cyril Chatain) upon reasonable request.

## Abbreviations

BMI: Body mass index
CI: Confidence interval
CMDT: Cognitive-motor dual-task
COPD: Chronic obstructive pulmonary disease
CTL: Control
CV: Coefficient of variation
DT: Dual task
FA: False alarms
FEV_1_: Forced expiratory volume in the first second
HR: Heart rate
ICC: Intraclass correlation
LMM: Linear mixed models
MCFS: Manchester chronic obstructive pulmonary disease fatigue scale
MD: Mean difference
MMSE: Mini-mental state examination
MVC: Maximal voluntary contraction
MVF: Maximal voluntary force
POMS: Profile of mood states
PSQI: Pittsburgh sleep quality index
PwCOPD: People with chronic obstructive pulmonary disease
ROF: Rating-of-fatigue scale
SD: Standard deviation
ST: Single task
TF: Task failure
TTF: Time to task failure
Tw_p_: Potentiated peak twitch
Tw_s_: Superimposed peak twitch
VAL: Voluntary activation level
VF_b_: Voluntary force produced just before superimposed peak twitch
